# Gastrointestinal Involvement in Lipoid Proteinosis: A Ten-Year Follow-Up of a Brazilian Female Patient

**DOI:** 10.1155/2014/952038

**Published:** 2014-06-19

**Authors:** Juliana Custódio Lima, Cristiane Kibune Nagasako, Ciro Garcia Montes, Irene Harumi Kamata Barcelos, Rita Barbosa de Carvalho, Maria Aparecida Mesquita

**Affiliations:** ^1^Gastroenterology Unit, Faculty of Medical Sciences, State University of Campinas (UNICAMP), 13083-887 Campinas, SP, Brazil; ^2^Gastrocenter, State University of Campinas (UNICAMP), 13083-878 Campinas, SP, Brazil; ^3^Department of Radiology, Faculty of Medical Sciences, State University of Campinas (UNICAMP), 13083-887 Campinas, SP, Brazil

## Abstract

Lipoid proteinosis is a rare autosomal recessive disease characterized by the deposition of hyaline material in the skin and internal organs. The main clinical features are hoarseness and typical skin lesions. In this report we describe the endoscopic and radiologic findings in a Brazilian female patient presenting extensive gastrointestinal involvement and the evolution of the detected lesions in ten years of follow-up. Initial upper endoscopy and colonoscopy showed a similar pattern of multiple yellowish nodules throughout the esophagus, stomach, duodenum, and colons. Histological analysis confirmed the diagnosis of lipoid proteinosis. In addition, small bowel follow through demonstrated numerous well defined, round, small filling defects throughout the jejunum. Ten years later, the esophageal lesions remained the same, but none of the previous alterations were detected in the stomach, duodenum, and colons. In conclusion, lipoid proteinosis may affect all gastrointestinal organs with the same pattern of macroscopic and microscopic lesions. Some lesions may regress with increasing age.

## 1. Background

Lipoid proteinosis (LP), also known as Urbach-Wiethe disease or hyalinosis cutis et mucosae, is a rare autosomal-recessive disease, with less than 400 cases reported to date [1–3]. The disorder is characterized by hyaline deposits in the skin, mouth, upper respiratory tract, and other viscera. The most common clinical features of LP are hoarseness, which may present in the first months of life, and skin lesions, characterized by skin infiltration and thickening, papules, chicken pox-like scars, and the presence of beaded papules along the eyelid [4]. 

Reports on gastrointestinal (GI) involvement in this condition are scarce. It appears that macroscopic lesions are unusual, although the histological analysis of visceral biopsies and autopsy specimens has shown the typical hyaline deposits in the esophagus, stomach, small bowel, and rectum [5]. Earlier reports on radiological studies, gastroscopic examination, and sigmoidoscopy in patients suffering from LP revealed no abnormality in the gastrointestinal tract [5, 6]. More recently, the involvement of the esophagus was documented in one patient from Saudi Arabia, but the authors provided no description of the endoscopic findings [7]. So far the only GI complication of the disease was described in one patient with acute gastrointestinal bleeding secondary to deposits typical of LP in the small bowel [8]. In all other cases patients had no gastrointestinal complaints. 

In this report we describe the typical macroscopic and histological characteristics of the lesions affecting the whole gastrointestinal tract in a Brazilian female patient with LP and the evolution of the detected lesions over a ten-year period.

## 2. Case Report 

A 20-year-old female patient attending the dermatology unit of our university hospital was referred to our gastroenterology outpatient clinic in 2002 due to symptoms of epigastric pain, postprandial fullness, and bloating. She reported no other upper or lower gastrointestinal symptoms.

The patient had a history of hoarseness since the first months of life. When she was one year old she presented with recurrent blisters, superinfected skin, and mucous membrane lesions that progressed to affect the whole body. Histological analysis of skin biopsies taken at the age of three showed deposition of eosinophilic hyaline PAS-positive material in the dermis and around small blood vessels, confirming the clinical diagnosis of LP. Her parents were nonconsanguineous and no other family members were affected.

The hoarseness progressed over the years, and she experienced recurrent superinfected blisters. The skin lesions healed after several treatments with different drugs, including etretinate and chloroquine, but visible scars developed in the areas where the lesions have been present. There was no history of seizures or neuropsychiatric abnormalities.

Previous laryngoscopy revealed thickening of the vocal cords and nodules on the base of the tongue and supraglottic portion. Magnetic resonance (MR) imaging showed symmetrical calcifications on the amygdaloid nucleus and uncus.

**Figure 1:**
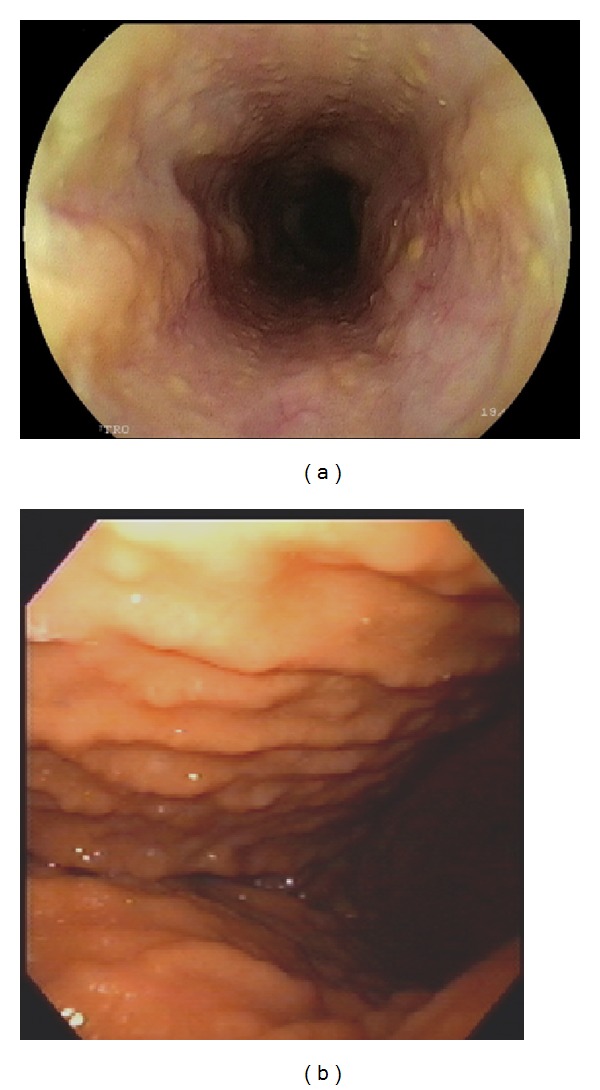
Upper endoscopy showing multiple yellowish nodules throughout the esophagus (a) and the body of the stomach (b).

**Figure 2:**
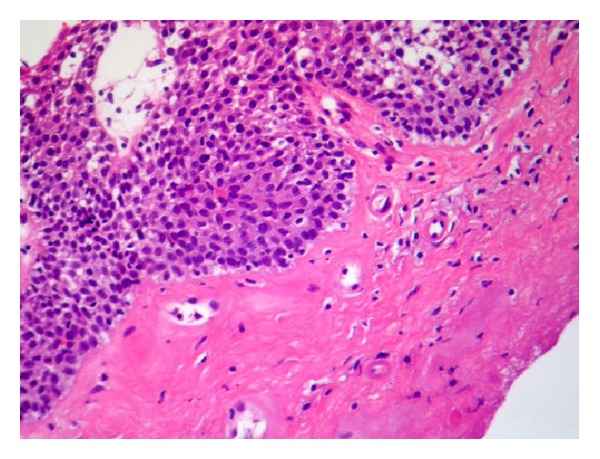
Histological analysis of esophageal biopsy showing deposition of eosinophilic hyaline material in the lamina propria and around blood vessels (hematoxylin and eosin stain).

On admission the patient had a hoarse voice, and multiple scars were visible on the face and body. Upper endoscopy showed multiple yellowish nodules throughout the esophagus (Figure 1(a)), body of the stomach (Figure 1(b)), and duodenum. There was no evidence of esophagitis or gastritis. Histological examination revealed deposition of eosinophilic hyaline material in the lamina propria and submucosa and around blood vessels (Figure 2). The material was positive on periodic acid-Schiff (PAS) staining and negative on Congo red staining, confirming the diagnosis of LP. Esophageal manometry was normal.

**Figure 3:**
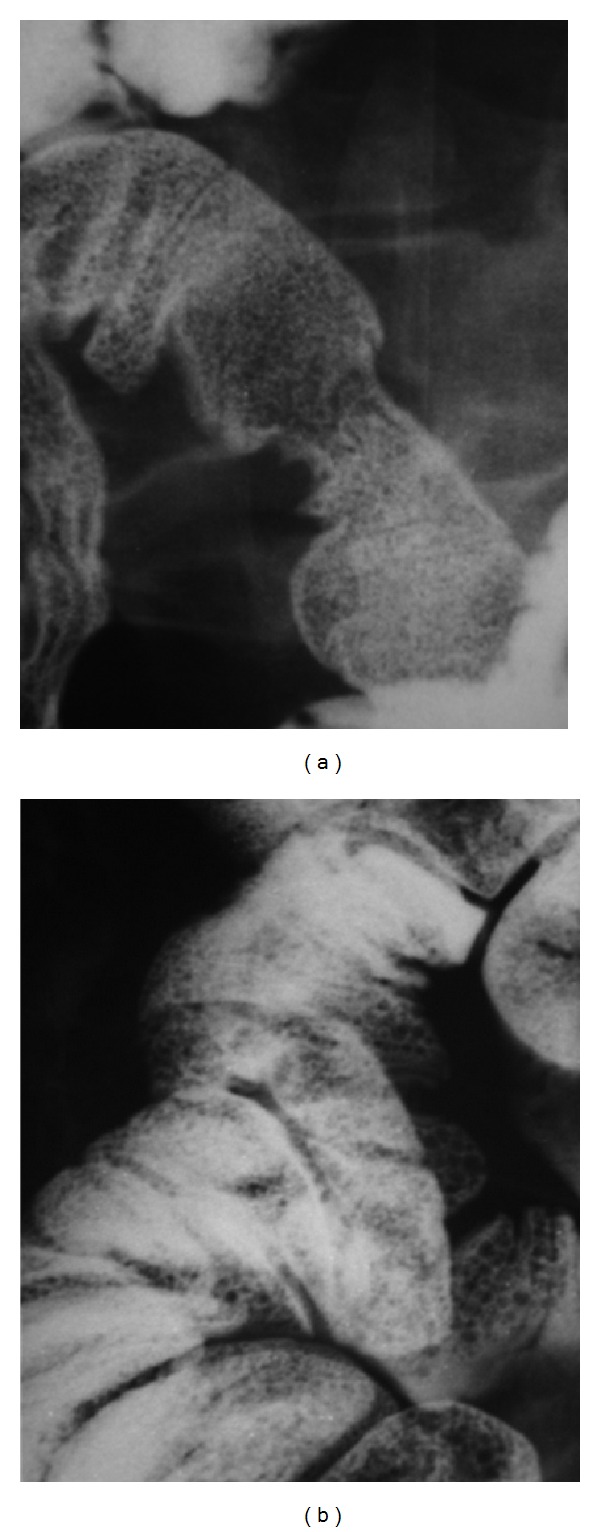
Small bowel follow through: multiple well defined, round, small filling defects all over the jejunum.

Colonoscopy also revealed multiple yellowish nodules measuring 0.2–0.4 cm distributed throughout the left colon. Small bowel follow through showed multiple well defined, round, small filling defects all over the jejunum and granularity of the mucosa along the ileum, with symmetrical fold pattern (Figure 3).

The patient received symptomatic treatment with Omeprazole at 40 mg/day and Domperidone at 10 mg before meals and reported a significant improvement after two months of therapy.

### 2.1. Ten-Year Follow-Up

During follow-up the patient experienced a few episodes of dyspepsia, successfully treated with the same medications. She had no other gastrointestinal symptoms during that period. The skin lesions and hoarseness remained stable and she developed no signal of neurological disease.

Upper endoscopy performed ten years after the initial examination showed the maintenance of the esophageal lesions. Histological analysis confirmed the typical deposition of PAS-positive hyaline material. In contrast, there was no evidence of the lesions previously detected in the stomach and duodenum. Likewise, no abnormality was found in the repeat colonoscopy.

## 3. Discussion

Molecular studies have shown that LP results from mutations in the extracellular matrix protein 1 gene* ECM1* [9, 10]. This protein is expressed in many tissues and has several biological functions, such as keratinocyte differentiation, binding of dermal collagens and proteoglycans, and regulation of angiogenesis.* ECM1* has binding sites for a number of extracellular matrix proteins and polysaccharides, including collagen IV, matrix metalloproteinase 9 (MMP-9), laminin, and perlecan [1, 11]. Therefore, the loss of function of* ECM1* may increase the expression of those proteins and result in impairment of the extracellular matrix structure.

The patient described in this report presented the typical features of LP. The disease started with hoarseness in the early childhood, she had the classical skin lesions, and the diagnosis was confirmed by skin histological examination. In addition, she presented cranial calcifications, which are also frequent in LP [12].

As far as we know, this is the first report describing the endoscopic and radiologic features of LP lesions affecting the whole GI tract, as well as the course of those alterations over a 10-year follow-up period.

The characteristic yellowish nodules were present in the esophagus, stomach, duodenum, and colon and probably in the small bowel, according to the radiologic findings. Histological analysis showed the typical deposition of eosinophilic PAS-positive hyaline material, similar to the findings in the skin biopsies, confirming the systemic character of LP in this patient. Our observations are in agreement with previous reports showing that in general gastrointestinal involvement in LP is asymptomatic [5].

Despite the persistence of LP lesions in the esophagus, the patient developed no esophageal symptom during follow-up. Moreover, the disappearance of the alterations in the stomach, duodenum, and colons indicates a relatively good prognosis of the disease in our patient. There is a lack of data regarding the course of LP. The assessment of oral manifestations in 27 patients suggested that the lesions may become more severe over the years [13]. In contrast, spontaneous improvement of skin lesions has been reported in one patient [2].

In conclusion, LP may affect all gastrointestinal organs with the same pattern of macroscopic and microscopic lesions. Our observations support previous remarks of a tendency for improvement with increasing age in some patients.
